# Supplementary material for: Controlling the thickness of the atherosclerotic plaque by statin medication
Source: PLoS One. 2020 Oct 13;15(10):e0239953. doi: 10.1371/journal.pone.0239953 (PMC7553348; doi:10.1371/journal.pone.0239953)
Supplement: Attachment — Submitted filename: Response to Reviewers.pdf [file pone.0239953.s001.pdf]

August 12, 2020

Dear Editor,

Re: *Controlling the thickness of the atherosclerotic plaque by statin medication*

Thank you for giving us an opportunity to revise and resubmit our paper. We are thankful to you and the referees for sharing your, and their, views about our research.

As per your request [—]:
Please include the following items [...]
• *A rebuttal letter that responds to each point raised by the academic editor and reviewer(s). You should upload this letter as a separate file labeled 'Response to Reviewers'.*

[—] we have combined our replies to your and Reviewer #1's comments and queries in this letter. Please find our replies below. Your comments and queries are in *italics.*

- *If applicable, we recommend that you deposit your laboratory protocols in protocols.io to enhance the reproducibility of your results.*

We think this recommendation is not applicable to our paper. Limited parameter estimation was performed and described in our previous paper cited as:

[9] Formanowicz D, Krawczyk JB, Perek B, Formanowicz P. 'A Control-Theoretic Model of Atherosclerosis.' *Int J Mol Sci.* 2019;20:785.

In the current paper, the parameters have been calibrated i.e., their values are proposed on the basis of other authors' research referenced to in our bibliography.

- *During our internal evaluation of the manuscript, we found significant text overlap between your submission and the following previously published works: https://www.mdpi.com/1422-0067/20/3/785/html*

  Please revise the manuscript to rephrase the duplicated text, cite your sources, and provide details as to how the current manuscript advances on previous work. Please note that further consideration is dependent on the submission of a manuscript that addresses these concerns about the overlap in text with published work.

The above mentioned paper has been also authored by us (plus two other coauthors). While writing the current paper we felt that some repetitions concerning, in particular, the representation of atherosclerosis as a dynamic process and the selection of the model variables, common for both papers, are unavoidable.
Action: We have tried and rephrased a significant amount of the overlapping text. See the green-highlighted parts in 'Revised Manuscript with Track Changes'.

- *Please clarify in your Data availability statement whether any existing datasets were used, and whether the code for the model has been made available.*

Action: Two Matlab files needed for production of Figures 3-5 are available from authors.

- *I do not understand how the viability kernels apply to their model. I would like to suggest writing the explicit formulation of viability kernels in the manuscript.*

The viability kernel in a controlled model with a target is the locus of states from which the target can be reached, using available controls. A reason for us to use the viability kernel in analysing atherosclerosis is that the medical problem of controlling patient's IMT to a desired thickness can be modelled and solved by computing the viability kernel, for a mathematical model of atherosclerosis progression.

We have said in the paper (in lines 30-31) that the "[..] two viability kernels [..] contain the atherosclerosis states, from which one can slow down the disease process". A more comprehensive description of the viability kernel is provided in the subsection "Viability kernels as 'safety regions' ", see lines 396-415.

We believe that a more mathematically involved description than we have provided could turn some medical specialists off form our model.

For interpretations, consider Figure 4. The clinician's goal, or target, is to let the patient live until 90 years with IMT below 1.15 [mm]. The green area is the set of patient's age and the corresponding IMT values, from which atherosclerosis - medicated by the statin therapy $s = 40[\texttt{mg/d}]$ - follows the evolution $x(t) \leq x^* = 1.15$ [mm] for $t \leq T^* = 90$ [yo]. So, the green area shows the clinician the disease states, from which the statin therapy $s = 40[\texttt{mg/d}]$ can control the disease to the target.

Figure 5, shows the disease states (see the yellow area that overlaps the green area) from which a stronger treatment, $s = 80[\texttt{mg/d}]$, can control the disease to the target.

Action: We have now included in the paper the yellow highlighted sentence from above, in lines 412-415.

- *In eq. 3, how can you guarantee the continuity at $t = T$?*

There is no need to assure this continuity. Eq (3) describes the derivative of c. We contend that the variable c describes patient's health status. The *pace* of c - i.e., the derivative - changes after a statin treatment is implemented. The treatment begins at T therefore the pace changes at T. This means that the derivative – $\frac{dc}{dt}$ – "jumps" at T. However, the integral of $\frac{dc}{dt}$ is c is continuous. This process is illustrated in Figure 3.

- *What's the dynamics of $s(t)$? it looks like you choose a constant in your simulation...*

This is correct: in the simulations, we use either $s = 40[\texttt{mg/d}]$ or $s = 80[\texttt{mg/d}]$.

There are many statin brands used in therapies, some will be stronger than some others. E.g., 80 [mg/d] of atorvastatin can be equivalent to 40 [mg/d] of pravastatin. The statins in our model can be identified as the latter i.e., stronger. The dose of $s = 80[\texttt{mg/d}]$ in our model is close to the typical maximum statin dose. Its half will be close to the typical starting dose. So, our selection of these two doses should be sufficient to understand our model's machinery.

As to the "dynamics" of $s(t) \in [40, 80]$, we note that $\frac{dc}{dt}$ is monotonic in s, see Eq (3), and so is its integral - $c(t)$. Therefore, if the statin dose - constant or variable - is restricted to $[40, 80]$, then the corresponding (to this statin dose) atherosclerosis evolution will remain between the evolutions emanating, respectively, from the points **A** and **B** in Figure 5.

Action: We have included in the paper a sentence to say what can happen when $s(t) \in (40, 80)$. See the yellow-highlighted text in lines 477-480.

- *How the viability kernels contribute to Eq. (3) and Eq. (1)?*

The relationship is the other way around: the kernels result from multiple solutions to the system of two differential equations: (1) and (3).

An atherosclerosis evolution (several such evolutions are shown as lines in Figures 4 and 5), is a solution to the system (1)-(3). The initial points of evolutions, which meet the target $x(T^*) \leq 1.15[\texttt{mm}]$, constitute the viability kernels.
